# Relation between temperature in the initial 24 hours in patients with severe sepsis or septic shock with mortality and length of stay in the ICU

**DOI:** 10.1186/cc10664

**Published:** 2012-03-20

**Authors:** R Sanga, S Zanotti, C Schorr, B Milcareck, K Hunter, P Dellinger, J Parrilo

**Affiliations:** 1 Cooper University Hospital, Camden, NJ, USA

## Introduction

Fever is a common event (ranges from 25 to 70%) in patients admitted to the ICU. The usual clinical approach in most units is to treat the fever either with medications (acetaminophen, nonsteroid anti-inflammatory drugs) or external measures, like cooling blankets. No studies assessed clinical evidence for these interventions. There is otherwise evidence that fever may be beneficial, inducing heat shock proteins and decreasing NK-κB activation. Also treating fever can mask an important clinical sign and avoid early treatment in patients with severe sepsis.

## Methods

We did a case-control study using two available databases and collected 750 patients with the diagnosis of severe sepsis and septic shock. We collected age, sex, days on mechanical ventilation, APACHE II score, vasopressor use and correlated with the presence of hyperthermia (>101.3°F), hypothermia (<96.8°F) and normothermia in the initial 24 hours. We used a mean of the available temperature data. Then we used logistic regression (univariate and multivariate) to compare these temperatures with mortality and length of stay in the ICU.

## Results

Compared to patients with normal temperature the hyperthermic patients had a lower mortality (22.58% vs. 39.1%) in the univariate analysis (*P *< 0.01). The patients with hypothermia had a mortality of 32.67% (NS). Length of stay was not significantly different between the groups. In the multivariate logistic regression the factors that were associated independently with mortality were age, APACHE II score, use of vasopressors, mechanical ventilation and temperature. Patients with *T *>101.3°F were 59% less likely to die when compared with patients with normal temperature.

## Conclusion

The results of this study highlight the importance of investigating the real effects of fever in severe sepsis or septic shock. Is it necessary to treat when they are not causing harm to the patients? Are we delaying diagnosis of severe sepsis because of the lack of this important clinical sign? The next step should be a prospective trial of treatment versus no treatment of fever in the ICU.

## References

[B1] Marik P Fever in the ICU Chest 2000 117 885 869 10.1378/chest.117.3.855 10713016

[B2] Levy M Clinical review of fever in intensive care unit patients Crit Care 2003 7 221 225 10.1186/cc1879 12793871 PMC270667

[B3] Dellinger P Surviving Sepsis Campaign Guidelines Crit Care Med 2008 36 296 327 10.1097/01.CCM.0000298158.12101.41 18158437

